# RGPDB: database of root-associated genes and promoters in maize, soybean, and sorghum

**DOI:** 10.1093/database/baaa038

**Published:** 2020-06-05

**Authors:** Gleb Moisseyev, Kiyoul Park, Alix Cui, Daniel Freitas, Divith Rajagopal, Anji Reddy Konda, Madalayne Martin-Olenski, Mackenzie Mcham, Kan Liu, Qian Du, James C Schnable, Etsuko N Moriyama, Edgar B Cahoon, Chi Zhang

**Affiliations:** 1 School of Biological Sciences, University of Nebraska, Lincoln, NE 68588 USA; 2 Department of Biochemistry, University of Nebraska, Lincoln, NE 68588 USA; 3 Department of Agronomy and Horticulture, University of Nebraska, Lincoln, NE 68583 USA; 4 Center for Plant Science Innovation, University of Nebraska, Lincoln, NE 68588, USA; 5 Young Nebraska Scientists Program, University of Nebraska (EPSCoR), Lincoln, NE 68588, USA

## Abstract

Root-associated genes play an important role in plants. Despite the fact that there have been studies on root biology, information on genes that are specifically expressed or upregulated in roots is poorly collected. There exist very few databases dedicated to genes and promoters associated with root biology, preventing effective root-related studies. Therefore, we analyzed multiple types of omics data to identify root-associated genes in maize, soybean, and sorghum and constructed a comprehensive online database of these genes and their promoter sequences. This database creates a pivotal platform capable of stimulating and facilitating further studies on manipulating root growth and development.

## Introduction

Roots are of critical importance for plant biology because they link below and above ground systems and extract water and nutrients from soil (1). The ability of roots to acquire minerals and water from the soil and to respond to the changing environments is dependent on the root system architecture, such as the root growth angle. It determines the direction of root elongation in the soil affecting the area in which roots capture water and nutrients. The root system and the root system architecture are the results of continuous root growth and development (2, 3). The root growth regulation is a highly complicated process and is controlled by complex gene interaction networks in both time and space. Identification of root-associated genes, their functions, and their interactions can reveal the physiological and molecular mechanisms that regulate the root growth and development and have the potential to improve crop production (4). Moreover, in plant biotechnology, synthetic promoters can provide precise spatial and temporal control of transgene expression to improve crop productivity (5, 6). The information of promoters in root-associated genes helps the synthetic biology tool development to generate plants with novel root traits to enhance plant performance (7, 8).

Due to the importance of roots, many root-associated genes have been studied. For example, some genes were discovered being involved in control of root-cell elongation in *Arabidopsis thaliana* mediated by the 1-aminocyclopropane-1-carboxylic acid (ACC) (9). Some others were found to have a function for regulation of root angle and gravitropism (10). Some root-associated genes were identified by genetic analysis of root response to drought stress and abscisic acid (11). Root-associated genes were also discovered and studied by gene expression profiling of the Arabidopsis root (12) and the Arabidopsis root transcriptome sequencing (13). These studies are, however, mainly for Arabidopsis. There are some but a limited number of studies on root-associated genes in maize, sorghum, and soybeans. For example, a few studies were conducted for genetic and genomic dissection of maize root development and architecture (14, 15). The expression of an expansin gene has been correlated with root elongation in soybean (16). For root-associated promoters, for example, *cis*-acting elements of the barley IDS2 gene promoter were found to confer iron-deficiency-inducible, root-specific expression in heterogeneous tobacco plants (17). However, there is no comprehensive data collection for root-associated genes and their promoters in maize, sorghum, and soybeans. Analysis of existing gene expression data facilitates the discovery of root-associated genes. Combining with genome sequence information, one can predict the promoter sequences for these candidate root-associated genes. Such information is useful for understanding and manipulating root growth and development.

Currently, there is only one root-associated gene related database—iRootHair (18). It includes information about 153 root hair-related genes that have been identified in dicots and monocots along with their putative orthologs in higher plants with sequenced genomes. There are some databases with information on *cis*-acting elements that control the transcription initiation by binding corresponding nuclear factors. They include TRANSFAC (19), JASPAR (20), TRANSCompel (21), PlantCARE (22) and PLACE (23). A more complete plant gene promoter database is PlantProm (24). However, the gene promoter data included in PlantProm are mainly for Arabidopsis. No databases have been developed specifically for root-associated genes and promoters, and especially for non-Arabidopsis plants.

To fulfill the important needs, we collected omics data and using these data, identified root-associated genes and their promoter sequences in maize, soybean, and sorghum. To maximize the value and usability of these types of data for efficient and effective data mining, we developed a web-based comprehensive database of root-associated genes and promoters. Our database, RGPDB, provides detailed information of both root-associated genes and the sequences of their promoter regions in maize, soybean, and sorghum.

**Figure 1:**
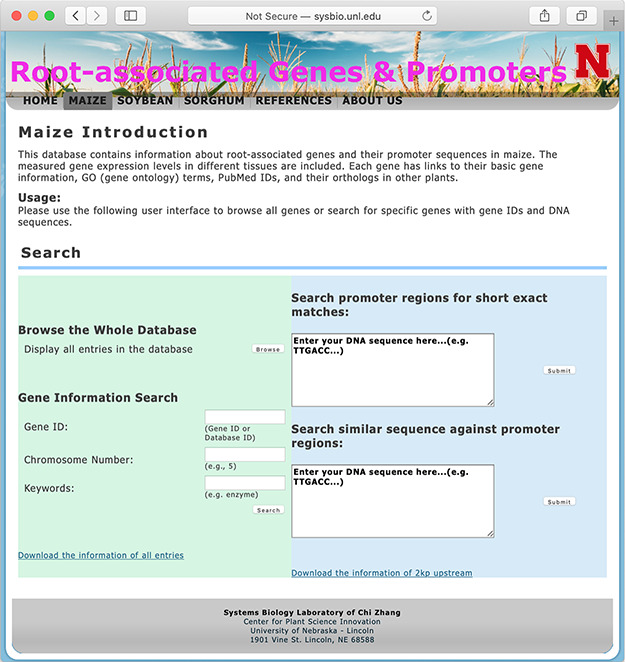
The searching page of the database.

## Data collection, root-associated gene prediction and validation

We collected multiple types of omics datasets for maize, soybean, and sorghum, including tissue transcriptomic and proteomic data. For transcriptomic data, data measured under different stresses were collected as well. More than 200 datasets were collected and analyzed. For maize genes, the following datasets were used: maize gene expression atlas by RNA-seq (25), a developmental atlas of maize (26), tissue-specific proteomics data (27), and gene expression in root with low nitrogen (28) and drought stress (29). For sorghum, RNA-seq data of Sorghum 9d seedlings in response to osmotic stress and abscisic acid (30), transcriptomics of sorghum tissues (31), gene expression profiles in root with nitrogen stress (32), and sorghum transcriptome database (33) were included. For soybean, transcriptomic data were mainly obtained from the collection in SoyBase (34), as well as from the RNA-Seq atlas of *Glycine max*, which has data for the soybean transcriptome in different tissues (35).

Root-associated genes were predicted as follows. An in-house tool, written in Perl, was used to integrate all gene expression data and identify root-associated gene candidates. To find a root-associated gene, we looked for a gene whose root expression level is at least 10 times larger than its maximal expression levels in all other tissues. If there are multiple studies for the root tissue, the maximal expression level of genes in all studies was used. When it was possible, protein products of the candidate genes and/or orthologs of these genes in other plants were examined. For example, maize proteomes data (27) were employed to validate candidate genes in maize to see if their protein products are also high in root. If the gene product is root-specific and its orthologs have high gene expression levels in roots as well, the corresponding gene is considered a high-confidence candidate. Orthologs of maize genes were obtained from MaizeGDB (36), Rice Genome Annotation Project (37), and References (38, 39). For soybean genes, their orthologs were obtained from OrthoDB (40) and the reference (41). Sorghum genes’ orthologs in Arabidopsis were obtained from Sorghum Functional Genomics Database (42), and orthologs in maize and rice are same for the maize data set. The gene expression atlas for other plants was obtained from various datasets, such as Schmid *et al.*’s work for Arabidopsis (43) and Rice Expression Database (44) for rice. For candidate genes, we extracted genomic sequences of the 2 kb regions upstream of a chosen TSS as promoter sequences because most transcriptional activity sets are within this region (45, 46). The information of each gene’s TSS was obtained from the gene annotation files. If there are multiple TSSs for a given gene, the position for the longest transcript was taken. If a gene has multiple alleles, only a single entry is included in the database.

To validate root-specific promoters that we identified in plant systems, four candidate maize genes were selected (*GRMZM2G027098*, *GRMZM2G477685*, *GRMZM2G125023* and *GRMZM2G133475*). Their upstream 2 kb regions were cloned and fused with β-glucuronidase (GUS) reporter gene and these *promoter: GUS* constructs were introduced into the rice. As shown in Figure 3, histochemical GUS data is in agreement with our prediction, suggesting that predicted maize gene promoters are subject to control by the root developmental process in monocots.

**Figure 2:**
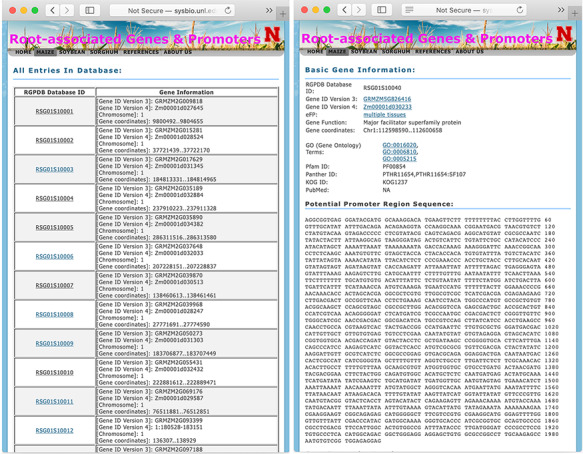
The display pages of searching result (left panel) and information of each gene (right panel).

**Figure 3:**
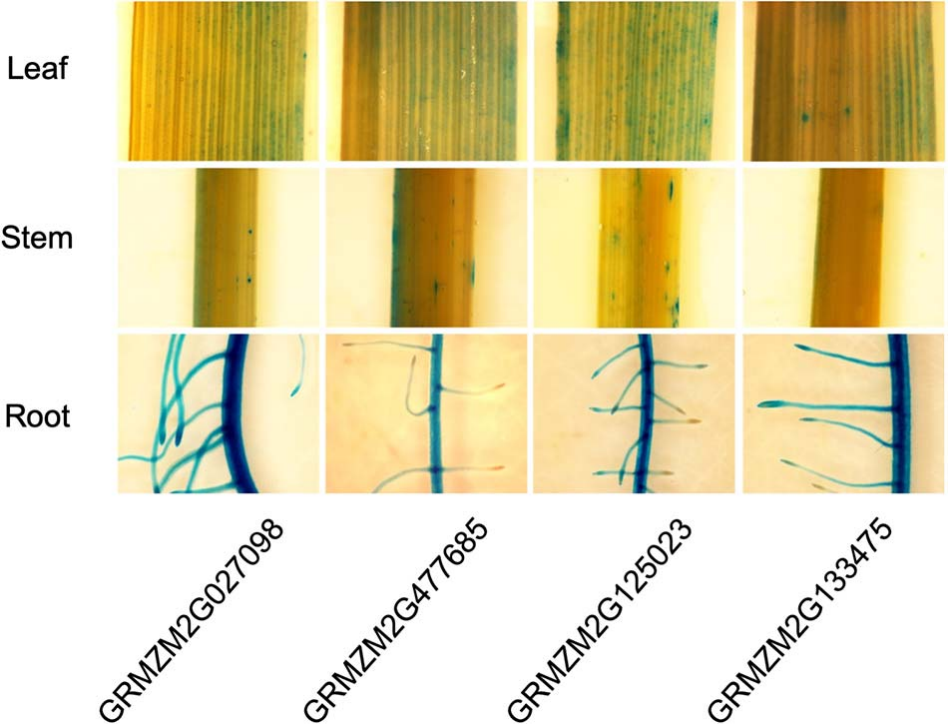
The histochemical GUS assay in a *promoter:GUS* transgenic rice.

## Database content

The current version of the database contains more than 1200 candidates of root-associated genes and their corresponding promoter sequences for maize (592), sorghum (363) and soybean (400). To store and query these data, we constructed an online database, RGPDB. In the online database, RGPDB, gene promoter sequences and other relevant resources are provided. For each gene, its normalized gene expression levels in different tissues are displayed, which were normalized by using DESeq package (47). These tissue-specific expression data can help users identify the most significant root-associated genes in the database for their interests. Other related information for each gene includes gene ID, description of the function, gene ontology (GO) annotations, Pfam IDs, orthologous genes in other plants and links to other databases, such as eFP browser (48), MaizeGDB (36) and SoyBase (34). For many genes, reference information (PubMed IDs) are also recorded. All information of a given gene can be downloaded as an XML or pdf file.

## User interface

### Browsing and searching

The database system provides interactive access to all of the collected data, and users may connect to the database using a web browser. Figure 1 shows a screen snapshot of the user interface for users to browse or search the database. The ‘browse’button allows users to get a list of all records in one table. Search options are provided to conveniently locate genes of interest by using, for example, gene IDs, RGPD Database IDs, chromosome numbers, keywords of gene annotations or DNA sequences of gene promoter regions.

### Displaying the database content

When a user browses the whole database or searches with a specific option, the database first returns a table of related records with RSG Database IDs and their annotations as shown in Figure 2. For maize, soybean, and sorghum genes, their RSG Database IDs start with RSG01S, RSG07S, and RSG05S, respectively. The detailed information of each gene can be displayed by clicking the link of their RGPD Database IDs. The information for each gene includes the basic information, promoter DNA sequences, GO annotation, Pfam ID, Panther ID, and their orthologs in various plant organisms. The basic information consists of gene IDs in different versions of the original database and function description. For maize, soybean, and sorghum genes, their gene IDs are linked to MaizeGDB (36), SoyBase (34) or Ensembl database (49), respectively. The PubMed information links to all related publications. If the protein coded by a gene has GO annotation, Pfam domain information, Panther domain information and/or EuKaryotic Orthologous Groups IDs, links to those domain IDs are provided. If a gene has orthologs from other plants, such as Arabidopsis or rice, the link to these orthologs’ databases and gene expression atlas databases are also provided, such as link to Rice Expression Database (44) for rice genes.

## Implementation

We adopted the LAMP (Linux, Apache, MySQL, PHP) platform to construct the online database system. The user interface additionally accepts parameters via a URL for direct searching. This feature facilitates a link to the database from external sites allowing users to bookmark and to cite directly specific results.

## Accessibility

The database is freely available to all users without restriction at https://crri.unl.edu/databases  and http://sysbio.unl.edu/RGPDB. The source codes and other detailed information are available upon request.

## Materials and Methods

### Rice transformation

Rice (*Oryza sativa* L.) japonica variety ‘Kitaake’ was used in this study. Transgenic rice plants were generated by Agrobacterium-mediated transformation method. Dry rice seeds were soaked in 70% ethanol for 3 min, treated with 0.3% NaClO solution for 20 min and rinsed with sterile water. Seeds were plated on MSD media (1x Murashige and Skoog (MS) medium including vitamins, 3% sucrose, 2 mg/ml 2, 4-D and 0.2% gelite, pH 5.8) to induce callus formation. Promoter-GUS vector-containing Agrobacterium was co-incubated with 7-day-old calli for 2 days on MSD media supplemented with 5% sorbitol and 200 M acetosyringone. After co-incubation, calli were rinsed extensively with distilled water containing 400 mg/l carbenicillin and plated on MSD media containing 30 mg/l hygromycin for selection. Selected calli were transferred to regeneration media (1x MS medium, 3% sucrose, 2% sorbitol, 2.5 mg/l kinetin, 0.1 mg/l NAA, 30 mg/ml hygromycin and 0.2% gelite, pH 5.8) for shooting. Plantlets that shoot were 3 ~ 5 cm long were transferred to rooting media (1x MS medium, 3% sucrose, 30 mg/ml hygromycin and 0.2% gelite, pH 5.8) and fully regenerated rice plants were transferred to the soil for further growth.

### Histochemical GUS assay

Transgenic rice plants were immersed in GUS staining buffer (50 mM sodium phosphate, 2 mM cyclohexyl ammonium salt, 0.5 mM K_3_Fe(CN)_6_ and 0.5 mM K_4_Fe(CN)_6_, pH 7.2) and incubated 8 h at 37°C with the dark condition. To destain, 70% ethanol were treated for 6 h.
